# Cardiotoxicity of chloroquine and hydroxychloroquine through mitochondrial pathway

**DOI:** 10.1186/s40360-023-00666-x

**Published:** 2023-04-21

**Authors:** Enayatollah Seydi, Mozhgan Karbalaei Hassani, Saghi Naderpour, Abdollah Arjmand, Jalal Pourahmad

**Affiliations:** 1 Department of Occupational Health and Safety Engineering, School of Health, Alborz University of Medical Sciences, Karaj, Iran; 2 Research Center for Health, Safety and Environment, Alborz University of Medical Sciences, Karaj, Iran; 3 Department of Toxicology and Pharmacology, School of Pharmacy, Shahid Beheshti University of Medical Sciences, Tehran, Iran; 4 Faculty of Pharmacy, Eastern Mediterranean University, Famagusta, North Cyprus Cyprus

**Keywords:** Chloroquine, Hydroxychloroquine, Cardiotoxicity, Mitochondria, Oxidative stress

## Abstract

**Background:**

Medical therapies can cause cardiotoxicity. Chloroquine (QC) and hydroxychloroquine (HQC) are drugs used in the treatment of malaria and skin and rheumatic disorders. These drugs were considered to help treatment of coronavirus disease (COVID-19) in 2019. Despite the low cost and availability of QC and HQC, reports indicate that this class of drugs can cause cardiotoxicity. The mechanism of this event is not well known, but evidence shows that QC and HQC can cause cardiotoxicity by affecting mitochondria and lysosomes.

**Methods:**

Therefore, our study was designed to investigate the effects of QC and HQC on heart mitochondria. In order to achieve this aim, mitochondrial function, reactive oxygen species (ROS) level, mitochondrial membrane disruption, and cytochrome c release in heart mitochondria were evaluated. Statistical significance was determined using the one-way and two-way analysis of variance (ANOVA) followed by post hoc Tukey to evaluate mitochondrial succinate dehydrogenase (SDH) activity and cytochrome c release, and Bonferroni test to evaluate the ROS level, mitochondrial membrane potential (MMP) collapse, and mitochondrial swelling.

**Results:**

Based on ANOVA analysis (one-way), the results of mitochondrial SDH activity showed that the IC_50_ concentration for CQ is 20 µM and for HCQ is 50 µM. Based on two-way ANOVA analysis, the highest effect of CQ and HCQ on the generation of ROS, collapse in the MMP, and mitochondrial swelling were observed at 40 µM and 100 µM concentrations, respectively (p < 0.05). Also, the highest effect of these two drugs has been observed in 60 min (p < 0.05). The statistical results showed that compared to CQ, HCQ is able to cause the release of cytochrome c from mitochondria in all applied concentrations (p < 0.05).

**Conclusions:**

The results suggest that QC and HQC can cause cardiotoxicity which can lead to heart disorders through oxidative stress and disfunction of heart mitochondria.

## Background

Various medical treatments can contribute to heart disease through cardiotoxicity. Chloroquine (CQ) and hydroxychloroquine (HCQ) are drugs used in the treatment of malaria. In addition, these drugs are used in the treatment of immune-mediated skin diseases and rheumatic diseases [1–3]. In recent years, CQ and HCQ have been used in the treatment of viral diseases, including coronavirus disease 2019 (COVID-19) [4, 5]. The difference between CQ and HCQ is a hydroxyl group and it is difficult to compare them. These drugs have the ability to accumulate in cells and organelles, including lysosomes [1, 6].

Reports have shown that CQ and HCQ can cause cardiotoxicity through unknown mechanisms, including disruption to the function of organelles such as lysosomes and mitochondria [7, 8]. These drugs are known as lysosomotropic agents and can accumulate in these organelles [1, 6, 9]. CQ and HCQ can increase the level of free radicals by the effect on respiratory chains in mitochondria and Fenton’s reaction in the lysosome. This event is associated with oxidative stress in the cell. Complexes I and III in the mitochondrial respiratory chain (MRC) are involved in the production of free radicals. In addition, these drugs can cause a collapse in the mitochondria membrane potential (MMP), the release of the cytochrome c from mitochondria to cytoplasm, and induce cell death through the mitochondrial pathway [5, 10–12].

Mitochondria play an important role in energy production, reactive oxygen species (ROS) production, and cell death as one of the most important organelles. The heart is one of the most important active organs in the body. This organ needs mitochondria to perform its normal functions. Accordingly, the number of mitochondria is abundant in the heart tissue, and it requires mitochondria as an important source of energy. Therefore, mitochondrial dysfunction is associated with heart disorders and the pathogenesis of heart diseases [13–15]. An increase in the level of ROS is directly related to the disruption of mitochondria, which results in damage to cellular components. In animal models, it has been reported that a reduction in ROS levels is associated with a reduction in cardiac disease [16–18]. Since the mechanism of cardiotoxicity caused by CQ and HCQ is not known, this study was designed to investigate the mechanism of cardiotoxicity of CQ and HCQ in heart mitochondria.

## Methods

### Animals

Male Wistar rats (180–220 g) were kept under standard laboratory conditions. All test methods were conducted according to the ethical standards and protocols approved by the Animal Experimentation Committee of Shahid Beheshti University of Medical Sciences, Tehran, Iran (Ethics code: IR.SBMU.PHARMACY.REC.1400.158).

### Heart mitochondria isolation

Cardiomyocytes have been used for mitochondrial isolation. At first, the perfusion technique was used to isolate cardiomyocytes [19–21]. Then, differential centrifugation was used for mitochondrial isolation from Cardiomyocytes. In the following, the centrifuge process was carried out in two stages: 1500 × g for 10 min at 4 °C, and 10,000 × g for 10 min at 4 °C. Eventually, the mitochondria were suspended in the corresponding buffers (Respiration, swelling, and MMP assay buffers) [20].

### Succinate dehydrogenase (SDH) evaluation

Assessment of SDH activity in heart mitochondria was done using MTT dye. To perform this experiment, mitochondrial suspension from the heart tissue was incubated with CQ (5, 10, 20, 40 µM) and HCQ (12.5, 25, 50, 100 µM) for 30 min at 37 °C. In the following, MTT dye was added to the mitochondrial suspension, and then dimethyl sulfoxide (DMSO) was used to dissolve the crystals caused by MTT dye. Eventually, the absorbance was assayed at 570 nm The IC_50_ concentration was 20 µM for CQ and 50 µM for HCQ [22].

### ROS level evaluation

ROS level evaluation in heart mitochondria was performed by 2’-7’dichlorofluorescin diacetate (DCFH-DA). To perform this experiment, heart mitochondria were incubated with CQ (10, 20, 40 µM) and HCQ (25, 50, 100 µM) in respiration assay buffer (10 mM Tris, 0.32 mM sucrose, 0.5mM MgCl_2_, 50 µM EGTA, 20 mM Mops, 5 mM sodium succinate and 0.1mM KH_2_PO_4_). After the incubation of the heart mitochondria with CQ and HCQ, DCFH-DA (10 µM) was added to the mitochondrial suspension. In the following, 5, 30, and 60 min after the incubation of the fluorescence intensity was evaluated using a spectrophotometer at wavelengths of E_x_: 488 nm and E_m_: 527 nm [20].

### Mitochondrial swelling

To evaluate mitochondrial swelling, heart mitochondria were suspended in swelling assay buffer (230 mM mannitol, 70 mM sucrose, 2 mM Tris–phosphate, 3 mM HEPES, 1 µM of rotenone, and 5 mM succinate), and then incubated with 2 CQ (10, 20, 40 µM) and HCQ (25, 50, 100 µM). In the following, 5, 30, and 60 min after the incubation, the absorbance was assayed at 540 nm using an ELISA reader. There is a direct correlation between decreased absorbance in samples and mitochondrial swelling [20].

### MMP collapse evaluation

MMP evaluation in heart mitochondria was performed by rhodamine 123 (Rh 123). To perform this experiment, heart mitochondria were incubated with CQ (10, 20, 40 µM) and HCQ (25, 50, 100 µM) in MMP assay buffer (10 mM KCl, 68 mM D-mannitol, 220 mM sucrose, 5 mM KH_2_PO_4_, 50 µM EGTA, 2 mM MgCl_2_, 5 mM sodium succinate, 2 mM Rotenone, and 10 mM HEPES). After the incubation of the heart mitochondria with CQ and HCQ, Rh 123 (10 µM) was added to the mitochondrial suspension. In the following, 5, 30, and 60 min after the incubation of the fluorescence intensity was evaluated using a spectrophotometer at wavelengths of E_x_: 490 nm and E_m_: 535 nm [23].

### Cytochrome c release evaluation

The effect on CQ (10, 20, 40 µM) and HCQ (25, 50, 100 µM) on cytochrome c release was assayed via using the Quantikine Human and Rat/ mouse Cytochrome c Immunoassay kit (R&D Systems Quantikine ELISA Kits, Minneapolis, MN, USA).

### Statistical analysis

Results were analyzed using GraphPad Prism 5 (GraphPad Software, La Jolla, CA). p < 0.05 were considered statistically significant.

## Results

### CQ/HCQ and SDH activity

**Fig. 1:**
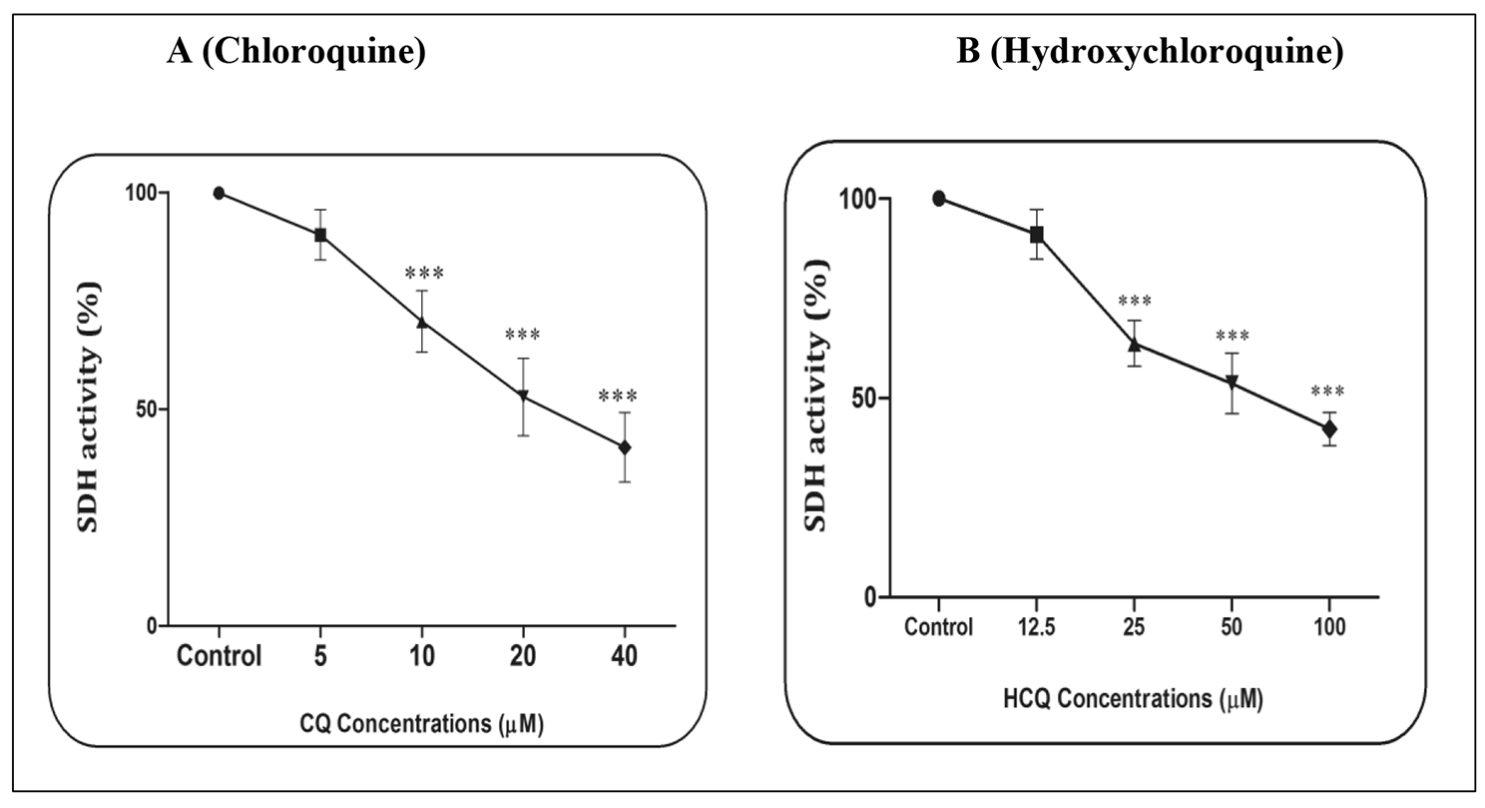
**SDH activity assay.** The effect of Chloroquine (A) and Hydroxychloroquine (B) on the SDH activity in the heart mitochondria. Data are presented as mean ± SD (n = 3). ^***^ (P < 0.001) shows a significant difference in comparison with control group

Based on a pilot study, the effects of CQ (5, 10, 20, and 40 µM) and HCQ (12.5, 25, 50, and 100 µM) were evaluated on mitochondrial function through SDH activity. The report showed that CQ (Fig. 1A) and HCQ (Fig. 1B) in all the concentrations used except the lowest concentration (p > 0.05) were able to reduce the activity of SDH (p < 0.001). This event is associated with a decrease in mitochondrial function (Fig. 1A-B). The results based on the one-way ANOVA test showed that CQ at a concentration of 40 µM in 60 min (p < 0.001) and HCQ at a concentration of 100 µM and in 60 min (p < 0.001) had the highest effect on reducing SDH activity. The IC_50_ concentration was 20 µM for CQ and 50 µM for HCQ.

### CQ/HCQ and ROS level

**Fig. 2:**
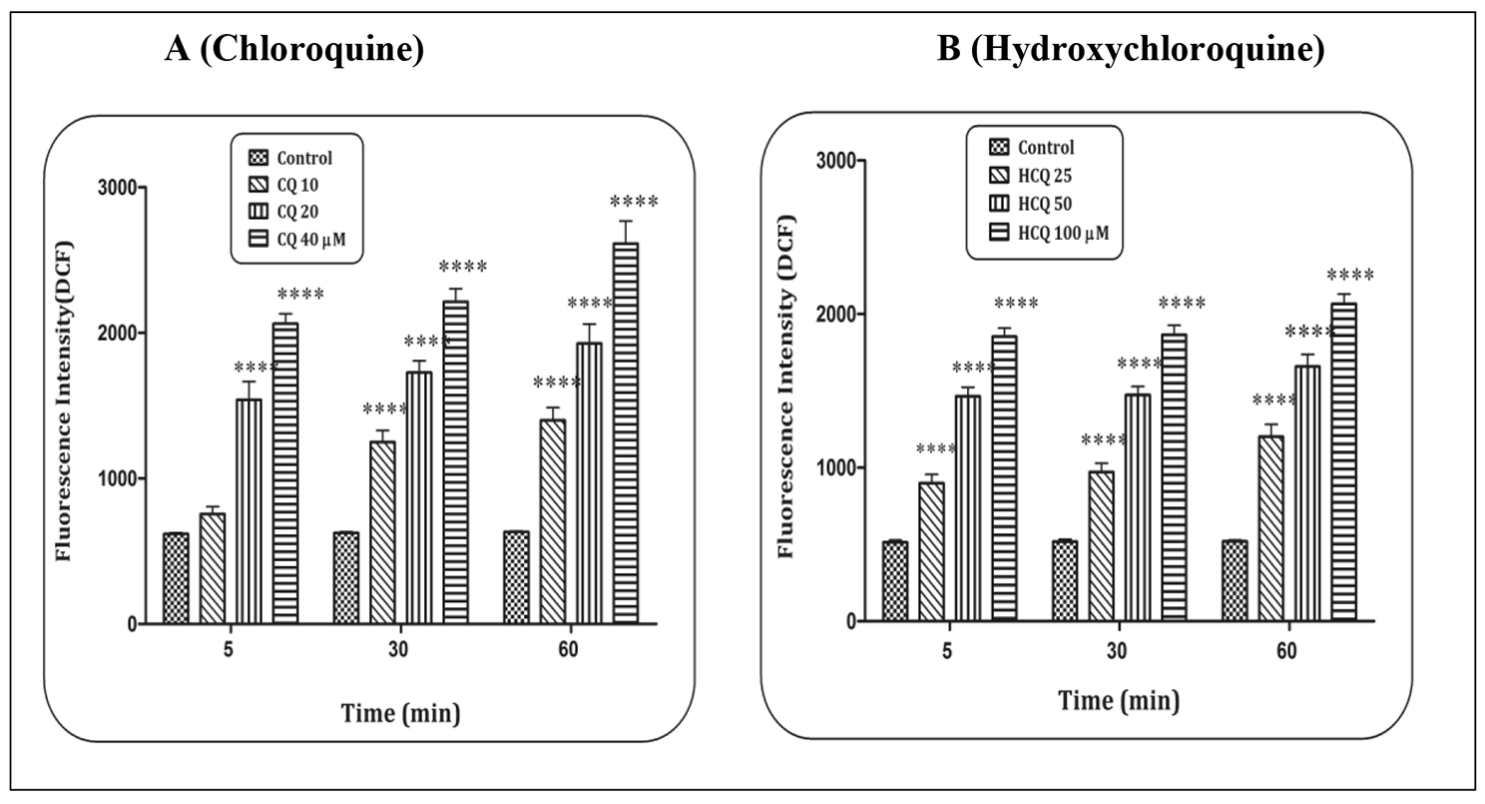
**ROS formation assay.** The effect of Chloroquine (A) and Hydroxychloroquine (B) on the ROS level in the heart mitochondria. Data are presented as mean ± SD (n = 3). ^****^ (P < 0.0001) shows a significant difference in comparison with control group

Based on the MTT test and IC_50_ concentration, concentrations of 10, 20 and 40 µM of CQ, and 25, 50, and 100 µM of HCQ were used to evaluate the level of ROS and other tests in mitochondria isolated from heart tissue. The results indicated that CQ (Fig. 2A) and HCQ (Fig. 2B) in a concentration-dependent pattern and at all exposure times (5, 30 and 60 min) were able to increase the level of ROS in heart mitochondria (p < 0.0001). Statistical reports showed that these two compounds at the highest concentration and the highest time have been able to significantly (p < 0.0001) increase the level of ROS in isolated mitochondria.

### CQ/HCQ and MMP collapse

**Fig. 3:**
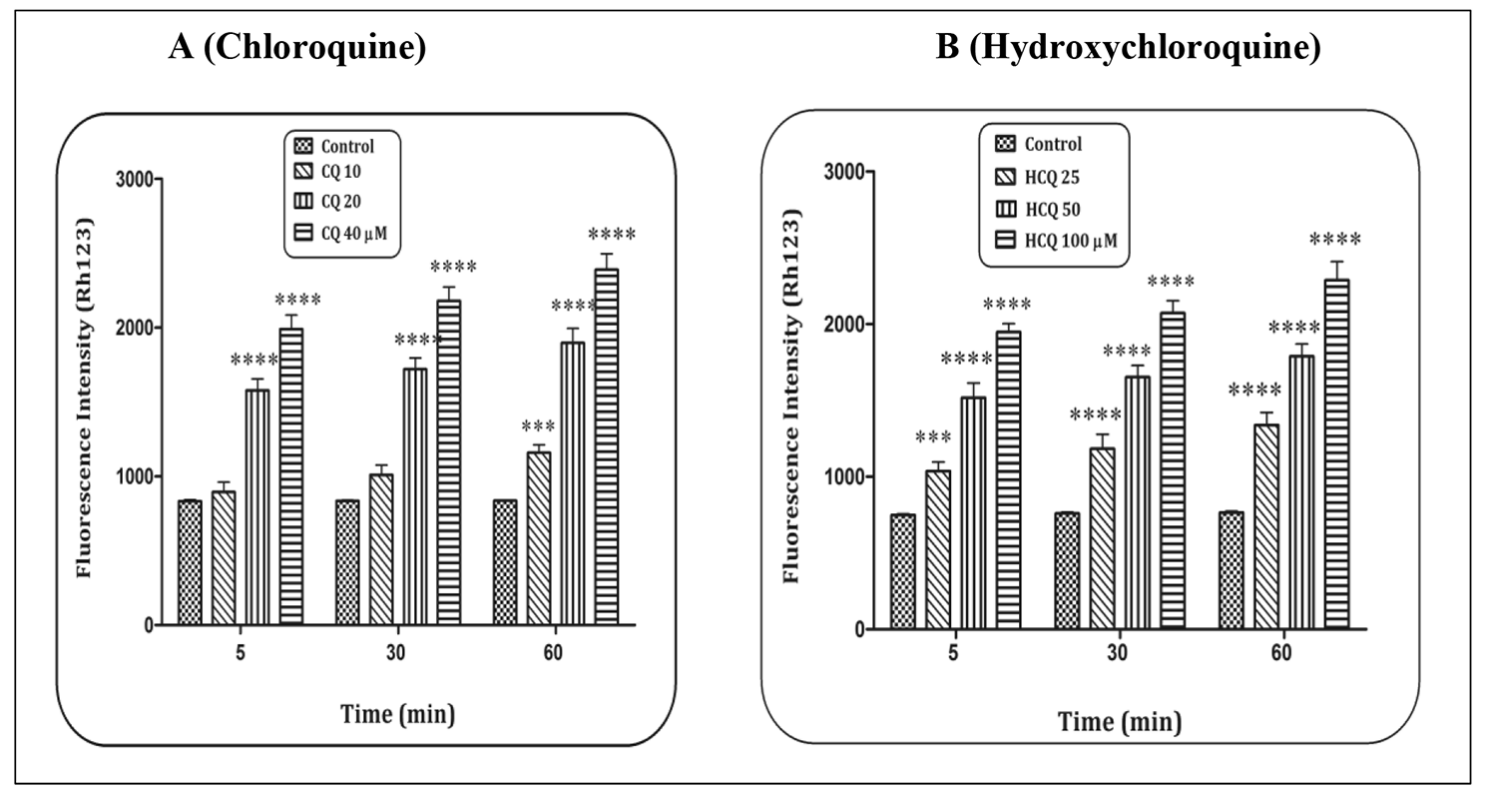
**Mitochondrial membrane potential (MMP) assay.** The effect of Chloroquine (A) and Hydroxychloroquine (B) on the MMP collapse in the heart mitochondria. Data are presented as mean ± SD (n = 3). ^***^ (P < 0.001) and ^****^ (P < 0.0001) shows a significant difference in comparison with control group

An increase in the level of ROS can be associated with the disruption of the mitochondrial membrane and the consequence of the collapse of the MMP. Accordingly, the effect of CQ and HCQ on MMP collapse in heart mitochondria was evaluated. The results showed that CQ at the lowest concentration was able to increase the level of ROS (60 min) in heart mitochondria (p < 0.001). However, in concentrations of CQ f 20 and 40 µM, this event has occurred in all exposure times (Fig. 3A) (p < 0.0001). Meanwhile, HCQ has increased the level of ROS in heart mitochondria in all concentrations used and exposure times (Fig. 3B) (p < 0.001 and p < 0.0001). The results showed that both compounds were able to cause a collapse in the MMP in a concentration- and time-dependent pattern.

### CQ/HCQ and mitochondrial swelling

**Fig. 4:**
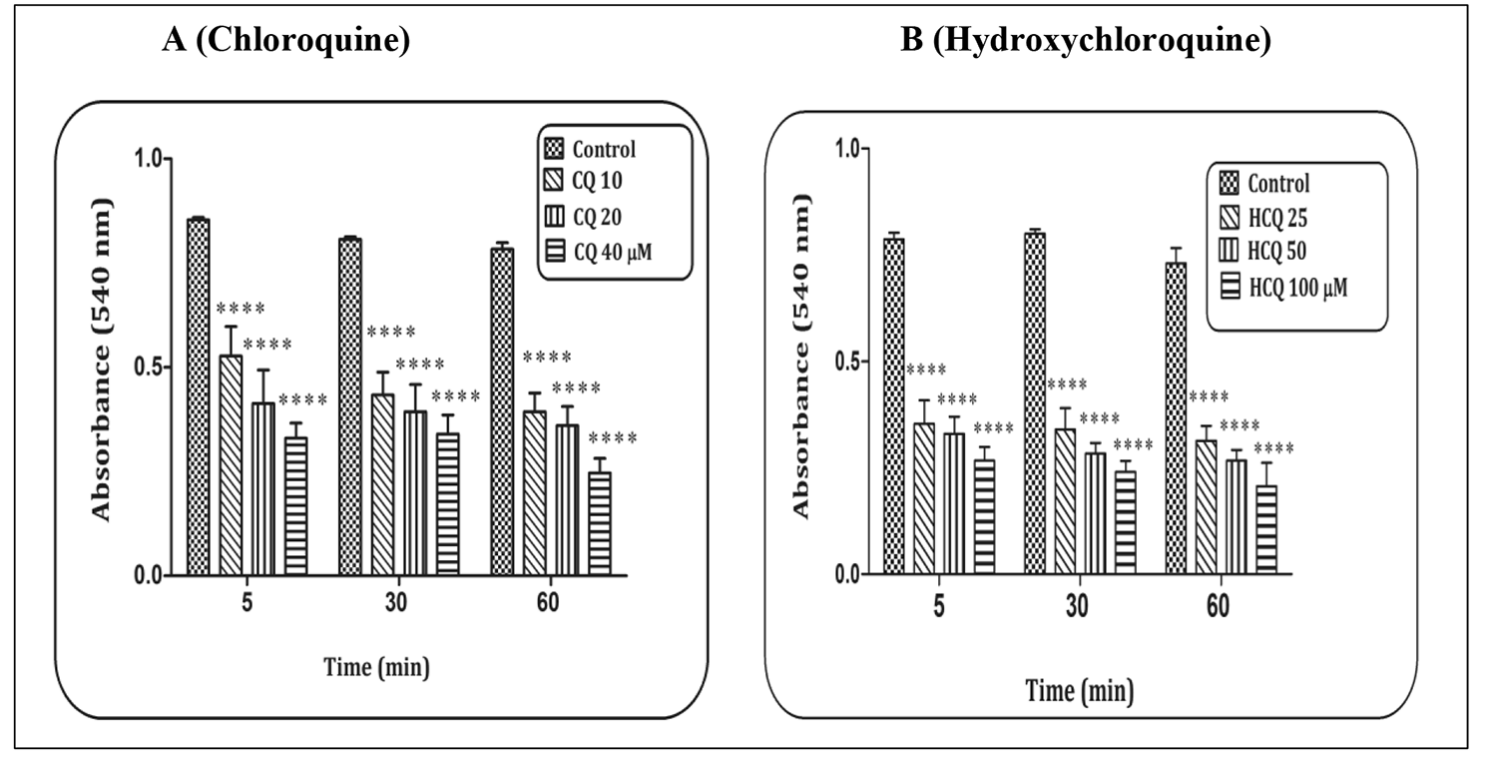
**Mitochondrial swelling assay.** The effect of Chloroquine (A) and Hydroxychloroquine (B) on the mitochondrial swelling in the heart mitochondria. Data are presented as mean ± SD (n = 3). ^****^ (P < 0.0001) shows a significant difference in comparison with control group

Mitochondrial swelling is known as another consequence of ROS production. The results indicated that CQ (Fig. 4A) and HCQ (Fig. 4B) in a concentration-dependent pattern and at all exposure times (5, 30, and 60 min) were able to increase the mitochondrial swelling in heart mitochondria (p < 0.0001). The highest rate of mitochondrial swelling occurred at the highest concentration and highest time.

### CQ/HCQ and cytochrome c release

**Fig. 5:**
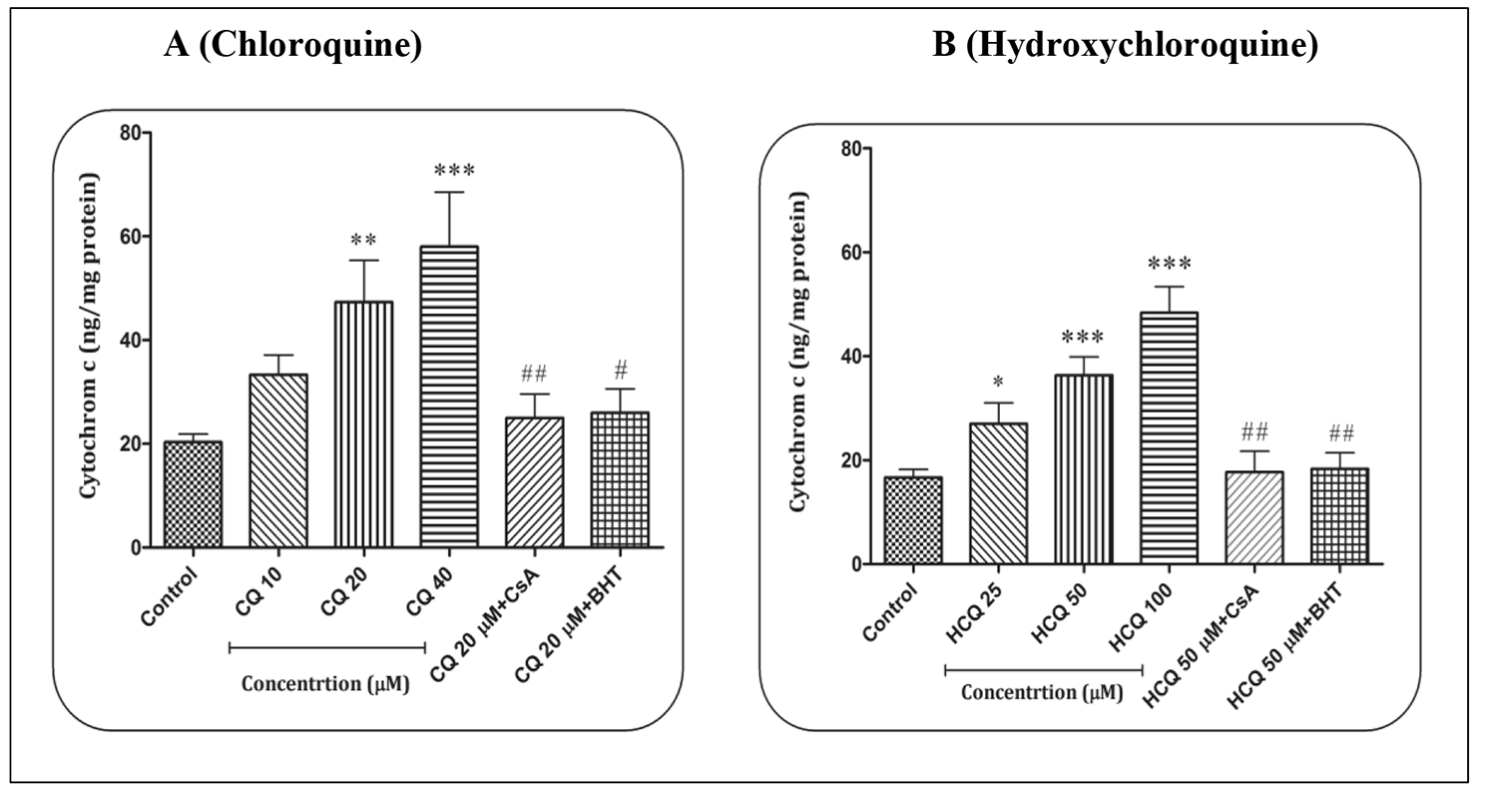
**Cytochrome c release assay.** The effect of Chloroquine (A) and Hydroxychloroquine (B) on the cytochrome c release in the heart mitochondria. Data are presented as mean ± SD (n = 3). ^*^ (P < 0.05), ^**^ (P < 0.01) and ^***^ (P < 0.001) shows a significant difference in comparison with control group. ^##^ (P < 0.01) shows a significant difference in comparison with Chloroquine (20 µM) and Hydroxychloroquine (50 µM) group

The report shows that CQ in concentrations of 20 (p < 0.01) and 40 µM (p < 0.001) and HCQ in concentrations of 25 (p < 0.05), 50 (p < 0.001) and 100 µM (p < 0.001) were able to cause the release of cytochrome c from heart mitochondria. The statistical results showed that compared to CQ, HCQ is able to cause the release of cytochrome c from mitochondria in all applied concentrations (p < 0.05), while this effect was not observed in the lowest concentration of CQ (p > 0.05). Cyclosporine A (Cs.A) as a mitochondrial permeability transition pore (mPTP) inhibitor and butylated hydroxytoluene (BHT) as an antioxidant were able to prevent the release of cytochrome c caused by CQ (20 µM) (p < 0.01 and p < 0.05, respectively) and HCQ (50 µM) (p < 0.01) in heart mitochondria (Fig. 5A-B).

## Discussion

Heart disorders are one of the major problems of public health [24]. In order to investigate the mechanism of cardiotoxicity caused by exposure to CQ and HCQ, parameters such as mitochondrial function, ROS level, mitochondrial membrane disruption and cytochrome c release were evaluated. Research has shown that QC and HQC can cause mitochondrial dysfunction [5, 11]. For the treatment of COVID-19, it was shown that HCQ and similar compounds have the ability to accumulate in mitochondria and can inhibit mitochondrial ATP production by disrupting the electrochemical proton gradient [25]. Therefore, HCQ can disrupt the tissues that need ATP for their proper activity. The results of another study showed that HCQ can cause mitochondrial abnormalities [26].

Mitochondria are known as the main intracellular source in the production of ROS. Production of ROS, decrease in mitochondrial content, decrease in the activity of complexes in the respiratory chain, and change in mitochondrial morphology are the most important characteristics of mitochondrial dysfunction [27]. In cardiomyocytes, the number of mitochondria is high for normal function. Furthermore, metabolism in the heart is aerobic and mitochondria provide 90% of the energy (ATP) required by cardiomyocytes [28, 29].

Initially, the results indicated that QC and HQC reduced the mitochondrial function/mitochondrial complex II in heart mitochondria. Complex II plays a role in the production of ROS. ROS plays a role in regulating important signaling pathways in the cell, some of which have cardioprotective effects [28]. In the heart, complexes I, II and, III in the MRC are the source of ROS production. The level of antioxidant enzymes in heart tissue is low and as a result, it is sensitive to high levels of free radicals (ROS). Therefore, the high level of ROS and the resulting oxidative stress can cause several complications in the heart [30, 31].

The study of Chaanine et al., showed that QC in high doses could cause cardiotoxicity in a rat model of pressure overload hypertrophy through the dysfunction of mitochondria and lysosomes. In this study, it has been shown that QC caused disruption in the antioxidant capacity of mitochondria and increased oxidative stress (ROS generation) [32]. Therefore, QC can provide conditions for oxidative stress. Oxidative stress is a destructive condition that can play a role in the pathophysiology of various diseases [33, 34]. The results showed that QC and HQC have been able to increase the level of ROS in heart mitochondria. The event is associated with oxidative stress and oxidative stress can initiate mitochondrial changes and consequently mitochondrial dysfunction [35, 36]. Previous studies have shown that QC and HQC can increase the level of ROS and cause oxidative stress [5, 12].

In the current research, the MMP collapse and mitochondria swelling were observed in heart mitochondria exposed to QC and HQC. Both events disrupt the mitochondria structure. Also, ROS can be the source of these two events. In heart defects, mitochondria are swollen and the density of the mitochondrial matrix has decreased [37]. In mitochondria, the MMP plays a driving force in energy (ATP) production. Also, it is involved in the induction of cell death [38]. Therefore, the MMP collapse is associated with irreparable consequences. Our results are in agreement with past studies that have shown that QC causes MMP collapse [6, 11, 12]. The release of cytochrome c is one of the consequences of the collapse in the MMP. In apoptosis signaling, the release of cytochrome c from mitochondria is known as one of the early events [39, 40]. Our results indicated that QC and HQC have led the release of cytochrome c from heart mitochondria. This is the result in agreement with previous studies that QC has led to the release of cytochrome c from the Mitochondria [10]. Due to the dependence of cardiomyocytes on mitochondria, dysfunction of mitochondria is associated with heart disorders [41, 42].

## Conclusion

Based on the results, it is suggested that QC and HQC increase the level of ROS and oxidative stress by affecting respiratory chain complexes in mitochondria. In addition, ROS levels increased by QC and HQC, causing a collapse in MMP and mitochondrial swelling. These events have been associated with the release of cytochrome c from heart mitochondria. Subsequently, QC and HQC can cause heart disorders by initiating mitochondrial apoptosis signaling in heart cardiomyocytes.

## Data Availability

The datasets used and analyzed during the current study are available from the corresponding author upon reasonable request.
